# Authors' reply

**Published:** 2010

**Authors:** Kalpana Babu, Vidya Satish, S Satish, D K SubbaKrishna, Mariamma Philips Abraham, Krishna R Murthy

**Affiliations:** Vittala International Institute of Ophthalmology & Prabha Eye Clinic and Research Center, Bangalore, India; 1 Santosh Diagnostic Centre, Bangalore, India; 2 Department of Biostatistics, National Institute of Mental Health Sciences, Bangalore, India

Dear Editor,

We wish to thank Chabblani[1] for their interest in this article.[2]

We would like to clarify a few points raised in their letter.

In all patients with a positive quantiferon TB gold test, a detailed systemic evaluation by a chest physician with special interest in tuberculosis followed up with basic laboratory investigations like Mantoux test, ESR, Chest X-ray and liver enzymes were done before labeling them false positives.

It has been mentioned in our article in the 'Materials and methods' that history of prior BCG vaccination could not be obtained in all cases due to inability to recall by the patient or absence of the BCG scar. It is also known that vaccine-induced reactions tend to wane over time and are unlikely to persist over 10 years.[3,4]

Group C had five out of 21 cases positive for the quantiferon TB gold test. Among the 21 cases in Group C, PPD positivity was seen in two cases. Only one case out of the 21 cases had both PPD and Quanti feron TB gold test positive while 15 of the 21 cases had both tests negative. In Group D, 28 cases out of 39 cases had both tests positive and had intraocular tuberculosis (response to antitubercular therapy). As a result, the specificity and sensitivity remains unchanged.
